# Post-ST-Segment Elevation Myocardial Infarction Follow-Up Care During the COVID-19 Pandemic and the Possible Benefit of Telemedicine: An Observational Study

**DOI:** 10.3389/fcvm.2021.755822

**Published:** 2021-10-22

**Authors:** Audrey A. Y. Zhang, Nicholas W. S. Chew, Cheng Han Ng, Kailun Phua, Yin Nwe Aye, Aaron Mai, Gwyneth Kong, Kalyar Saw, Raymond C. C. Wong, William K. F. Kong, Kian-Keong Poh, Koo-Hui Chan, Adrian Fatt-Hoe Low, Chi-Hang Lee, Mark Yan-Yee Chan, Ping Chai, James Yip, Tiong-Cheng Yeo, Huay-Cheem Tan, Poay-Huan Loh

**Affiliations:** ^1^Department of Cardiology, National University Heart Centre, National University Health System, Singapore, Singapore; ^2^Yong Loo Lin School of Medicine, National University of Singapore, Singapore, Singapore; ^3^Department of Medicine, National University Hospital, Singapore

**Keywords:** COVID-19, telemedicine, telehealth, ST-segment elevation myocardial infarction, pandemic

## Abstract

**Background:** Infectious control measures during the COVID-19 pandemic have led to the propensity toward telemedicine. This study examined the impact of telemedicine during the pandemic on the long-term outcomes of ST-segment elevation myocardial infarction (STEMI) patients.

**Methods:** This study included 288 patients admitted 1 year before the pandemic (October 2018–December 2018) and during the pandemic (January 2020–March 2020) eras, and survived their index STEMI admission. The follow-up period was 1 year. One-year primary safety endpoint was all-cause mortality. Secondary safety endpoints were cardiac readmissions for unplanned revascularisation, non-fatal myocardial infarction, heart failure, arrythmia, unstable angina. Major adverse cardiovascular events (MACE) was defined as the composite outcome of each individual safety endpoint.

**Results:** Despite unfavorable in-hospital outcomes among patients admitted during the pandemic compared to pre-pandemic era, both groups had similar 1-year all-cause mortality (11.2 vs. 8.5%, respectively, *p* = 0.454) but higher cardiac-related (14.1 vs. 5.1%, *p* < 0.001) and heart failure readmissions in the pandemic vs. pre-pandemic groups (7.1 vs. 1.7%, *p* = 0.037). Follow-up was more frequently conducted via teleconsultations (1.2 vs. 0.2 per patient/year, *p* = 0.001), with reduction in physical consultations (2.1 vs. 2.6 per patient/year, *p* = 0.043), during the pandemic vs. pre-pandemic era. Majority achieved guideline-directed medical therapy (GDMT) during pandemic vs. pre-pandemic era (75.9 vs. 61.6%, *p* = 0.010). Multivariable Cox regression demonstrated achieving medication target doses (HR 0.387, 95% CI 0.164–0.915, *p* = 0.031) and GDMT (HR 0.271, 95% CI 0.134–0.548, *p* < 0.001) were independent predictors of lower 1-year MACE after adjustment.

**Conclusion:** The pandemic has led to the wider application of teleconsultation, with increased adherence to GDMT, enhanced medication target dosing. Achieving GDMT was associated with favorable long-term prognosis.

## Introduction

The coronavirus-2019 (COVID-19) pandemic has demanded the rapid adaptation of healthcare operations in implementing measures to reduce the infectious rate but to also maintain the standard of patient care. Patients with cardiovascular disease are at increased risk of contracting the COVID-19 infection with a poorer outcome (1). The universally adopted strategy of social distancing as a measure to “flatten the curve” have resulted in a decrease in traditional physical consultations and the wider adaptation of teleconsultations. Teleconsultations, or telemedicine in general, offers virtual clinic consultations and monitoring which has gained traction as appropriate viable alternative for safe and efficient medical care. Its role has gained attention given the benefits of removing the risk of hospital exposure for these vulnerable patients during the pandemic. As the application of telemedicine expands, it becomes increasingly important to understand its impact on patient care and clinical outcomes.

During the pandemic, there has been a substantial reduction in patients presenting with ST-segment elevation myocardial infarction (STEMI) requiring primary percutaneous coronary intervention (PPCI) compared to the pre-pandemic era (2). Despite the decrease in PPCI case volume, the opposite effect of worse overall in-hospital STEMI performance metrics and short-term clinical outcomes were observed during the pandemic (3, 4). At present, little is known about the follow-up care of these STEMI patients during the pandemic and the potential role of telemedicine in the management of such patients following hospital discharge. This study is the first to examine the trend in teleconsultations for post-STEMI patients during the pandemic, and its association with optimal medical therapy, target medication doses, cardiovascular risk factor control and long-term clinical outcomes.

## Methods

### Setting and Design

This is a retrospective single-center study of patients with STEMI who presented to a major PCI-capable hospital in Singapore, and survived the index STEMI admission. Consecutive patients were enrolled into two study groups according to the date of their index admission: (1) Pre-pandemic, from 1 October 2018 to 31 December 2018, and (2) pandemic, from 1 January 2020 to 31 March 2020. Those who did not survive the index admission were excluded from the study. There were no patients who were admitted during both study periods. The follow-up was 1 year following the index STEMI admission. For at least 1 year post-STEMI, the cardiologists of the center visit would traditionally follow up with these patients closely whilst on dual-antiplatelet therapy. It was highly unlikely for these patients to be followed up by other cardiologists outside of the center visit, although these patients might be followed up by doctors from other sub-specialties based on their comorbidities. The time period for the pre-pandemic group was carefully chosen to allow a control with the closest temporal proximity to the COVID-19 pandemic period, without its 1-year post-STEMI follow-up being affected by the pandemic.

During the pandemic, particularly when the Disease Outbreak Response was heightened to its second highest level on 7 February 2020, the standard post-STEMI care after hospital discharge had to be rapidly revamped with increased adaptation of telemedicine. This involved virtual consultations that were conducted via a secure audio-visual telecommunication system between the patients and healthcare providers. Patients were encouraged to subscribe to the hospital telemedicine service and were either provided with or used their own equipment to measure blood pressure, pulse rate and body weight. Patients were also offered remote vital signs monitoring conducted daily for 1 month post-STEMI. Prescriptions were optimized based on the virtual assessment and delivered to the patient's homes. The main goal of teleconsultation during the COVID-19 pandemic was not to provide superior care to the standard face-to-face consultations, but to provide these patients with “health maintenance strategy” individualized to their needs and risk factor control targets (5, 6). The teleconsultation integrated virtual consultations, symptomology assessment, evaluation of home monitoring vitals such as blood pressure, patient education, drug tolerance and adherence, quality of life, and anticoagulation tolerance (7). Physical face-to-face consultations were still conducted, albeit less frequently, during the pandemic and these consultations involved serum testing for cardiovascular risk factor control. Serum measurements of glycated A1c (HbA1c), low-density lipoprotein (LDL) cholesterol, creatinine, estimated glomerular filtration rates (eGFR) and international normalized ratio (INR) (as appropriate) were taken during the physical consultations. Hence, these study periods were carefully chosen to compare the effectiveness of telemedicine on post-STEMI care during the pandemic, vs. the standard post-STEMI care during the pre-pandemic era. Patients with recurrent STEMI presentations during subsequent study periods were excluded to avoid duplication. During the pandemic, the hospital was actively involved in the care for COVID-19 patients.

None of the patients in the study were diagnosed with COVID-19. In our institution, the COVID-19 patients would be co-managed by the pandemic and the Cardiology inpatient teams. Once the COVID-19 patients have been de-isolated with negative COVID-19 polymerase chain reaction tests, they will be transferred under the Cardiology team's care. All patients, regardless of the COVID-19 status, will be reviewed outpatient in the Cardiology clinics. The COVID-19 status of the patients do not have any implications on their post-STEMI management.

### Data Collection

Data on demographic and clinical characteristics were retrospectively collected from the hospital STEMI registry. This included past medical history, cardiovascular risk factors, presentation type, presentation route, complications during index admission, and medications on discharge. Angiographic data were also collected from the electronic medical records. Follow-up outpatient data on the number of outpatient consultations (including physical consultations, teleconsultations and cardiac rehabilitation), remote vital signs monitoring uptake, reported symptoms in clinic, and post-discharge medications were obtained. Serial measurements of Hba1c, LDL, and systolic blood pressure during the follow-up period were collected.

Guideline-directed medical therapy for STEMI was defined as being on dual antiplatelet therapy (aspirin and P2Y12 inhibitor), statin, β-blocker, with the option of angiotensin-converting enzyme inhibitor or angiotensin receptor blocker (ACEI/ARB) if the post-STEMI left ventricular ejection fraction (LVEF) was ≤ 40% or the patient had diabetes mellitus (8, 9), unless these medications were clinically contraindicated in the individual. Patients who were on oral anticoagulation had to complete a month of triple antithrombotic therapy followed by concomitant oral anticoagulation and single antiplatelet, to be considered as being on guideline-directed medical therapy. β-blocker and angiotensin-converting enzyme inhibitors (ACEI) or angiotensin II receptor blocker (ARB) doses were recorded on discharge and at follow-up clinic. Achieving target dose intensity of β-blocker and ACEI/ARB was based on the type and dose of the medication in accordance to a standardized algorithm as defined by our previous study (10). Guideline-directed medical therapy at follow-up was recorded in any of the outpatient clinic visits during the first year post-STEMI. The presence of guideline-directed medical therapy during the outpatient follow-up was used for the multivariable analyses. Our institution adopted the protocol for dual antiplatelet therapy in accordance to the European Society of Cardiology (11) and American College of Cardiology/American Heart Association (12) guidelines in administering a potent P2Y_12_ inhibitor (prasugrel or ticagrelor), or clopidogrel if these are unavailable or contraindicated, and is usually prescribed before percutaneous coronary intervention is performed. Dual antiplatelet therapy was maintained over 12 months unless contraindicated.

### Study Outcomes

All study outcomes were measured during the 1-year follow-up from the discharge date of the index admission. The primary safety endpoint was all-cause mortality. Secondary safety endpoints were cardiac readmissions for unplanned revascularisation, non-fatal MI, heart failure, arrhythmia, unstable angina, and major adverse cardiovascular events (MACE). MACE was defined as the composite outcome of each individual safety endpoints.

Secondary efficacy outcomes measured were (1) prescription of guideline-directed medical therapy, (2) achieving target dose intensities of β-blocker and ACEI/ARB (10), and (3) cardiovascular risk factor control (systolic blood pressure, LDL, and HbA1c).

### Statistical Analyses

Categorical variables were described as percentages and continuous variables as mean with standard deviation (SD). Continuous variables were assessed with one-way analysis of variance (ANOVA). Categorical variables were evaluated with Pearson's chi-square test (or Fisher's Exact Test where appropriate). The multivariable Cox regression model was constructed to evaluate the association of telemedicine and 1-year MACE, as well as telemedicine and all-cause mortality, which included variables such as achieving medication target doses, guideline-directed medical therapy, remote vital signs monitoring, age, diabetes mellitus, chronic kidney disease, LVEF, smoking status, admission in the pandemic era, and presented with out-of-hospital cardiac arrest and/or cardiogenic shock. These co-variates were carefully chosen as they are traditional prognostic factors in STEMI patients.

Furthermore, *post-hoc* logistic regression was performed to evaluate the association of telemedicine and achieving guideline-directed medical therapy or medication target doses, which included co-variates such as age, smoking status, admission in the pandemic era, out-of-hospital cardiac arrest and cardiogenic shock, LVEF, gender, ethnicity, and presence of symptoms post-discharge. A p-value of < 0.05 was considered statistically significant. All statistical analyses were performed using IBM SPSS Statistics for Windows, Version 25.0. Armonk, NY. This study was conducted in accordance to the revised Declaration of Helsinki and approved by the institutional and local ethics committee (NHG DSRB No. 2013/00442). As the study involved retrospective analysis of clinically acquired data, the institutional review board waived the need for written patient consent.

## Results

### Baseline Characteristics

Table 1 displays the baseline characteristics of the study population. A total of 320 patients with STEMI who underwent primary PCI were reviewed retrospectively from the local STEMI registry. A total of 17 patients were lost to follow-up, with 6 patients from the pre-pandemic era and 11 from the pandemic era. All patients included in the analysis completed 1-year of follow-up. There were 15 inpatient deaths, 9 and 6 of whom were from the pandemic and pre-pandemic eras, respectively. After excluding inpatient deaths in the index hospitalization, 288 patients who survived their index admission were recruited in the study analysis. There were 170 (59.0%) STEMI patients in the pandemic group, and 118 (41.0%) in the pre-pandemic group. Baseline demographic characteristics and past medical history were similar between both groups. There were more evolved MI (13.5% vs. none, *p* < 0.001) and out-of-hospital cardiac arrest (6.5 vs. 5.1%, *p* < 0.001) in the pandemic group compared to the pre-pandemic group. Those who were admitted during the pandemic had higher incidence of unfavorable inpatient clinical progress compared to those admitted during the pre-pandemic era, such as Killip class 3 heart failure (15.3 vs. 6.8%, *p* = 0.028), sepsis (10.7 vs. 4.2%, *p* = 0.049), new onset atrial fibrillation (8.2 vs. 1.7%, *p* = 0.017) and lower LVEF (44 vs. 49%, *p* = 0.002). Importantly, there was no difference in discharge medications between the pandemic and pre-pandemic groups, apart from P2Y12 inhibitor use (100 vs. 94.1%, *p* = 0.001, respectively). Of the 7 patients discharged without P2Y12 inhibitor, only 1 was on concomitant oral anticoagulation with aspirin. The prescription of guideline-directed medical therapy on discharge between both groups was similar (*p* = 0.748).

**Table 1:** Baseline characteristics of study participants with ST-segment elevation myocardial infarction during index admission according to pre-pandemic or pandemic era.

	**Total (***n*** = 288)**	**Pandemic (***n*** = 170)**	**Pre-pandemic (***n*** = 118)**	***P*** **-value**
**Demographic**				
Age, years	59 (13)	59 (13)	58 (12)	0.626
Sex, female	46 (16.0)	29 (17.1)	17 (14.4)	0.546
Ethnicity				0.448
Chinese	142 (49.3)	88 (51.8)	54 (45.8)	
Malay	58 (20.1)	29 (17.1)	29 (24.6)	
Indian	66 (22.9)	39 (22.9)	27 (22.9)	
Other	22 (7.6)	14 (8.2)	8 (6.8)	
**Medical history**				
Smoking status				0.952
Non-smoker	130 (45.1)	78 (45.9)	52 (44.1)	
Active smoker	124 (43.1)	72 (42.4)	52 (44.1)	
Ex-smoker	34 (11.8)	20 (11.8)	14 (11.9)	
Hypertension	169 (58.7)	98 (57.6)	71 (60.2)	0.669
Diabetes	113 (39.2)	70 (41.2)	43 (36.4)	0.418
Hyperlipidaemia	179 (62.2)	100 (58.8)	79 (66.9)	0.162
Previous myocardial infarction	38 (13.2)	20 (11.8)	18 (15.3)	0.389
Previous PCI	45 (15.6)	21 (12.4)	24 (20.3)	0.066
Previous CABG	5 (1.7)	1 (0.6)	4 (3.4)	0.073
Stroke	14 (4.9)	8 (4.7)	6 (5.1)	0.883
Chronic kidney disease	23 (8.0)	15 (8.8)	8 (6.8)	0.529
Atrial fibrillation	8 (2.8)	4 (2.4)	4 (3.4)	0.598
Previous heart failure	9 (3.1)	6 (3.5)	3 (2.5)	0.636
Family history of premature CAD	37 (12.8)	28 (16.5)	9 (7.6)	**0.027**
**Index admission**				
Presentation type				**<0.001**
STEMI	248 (86.1)	136 (80.0)	112 (94.9)	
Evolved MI	23 (8.0)	23 (13.5)	0	
Out-of-hospital cardiac arrest	17 (5.9)	11 (6.5)	6 (5.1)	
Presentation route				0.156
Direct visit	199 (69.1)	112 (65.9)	87 (73.7)	
Interhospital transfers	89 (30.9)	58 (34.1)	31 (26.3)	
Complications				
Heart failure (Killip class 3)	34 (11.8)	26 (15.3)	8 (6.8)	**0.028**
Sepsis	23 (8.0)	18 (10.7)	5 (4.2)	**0.049**
New onset atrial fibrillation	16 (5.6)	14 (8.2)	2 (1.7)	**0.017**
Major bleed	27 (9.4)	18 (10.6)	9 (7.6)	0.397
Cardiogenic shock	21 (7.3)	13 (7.6)	8 (6.8)	0.781
Stroke	3 (1.0)	3 (1.8)	0	0.147
Acute kidney injury	53 (18.4)	27 (15.9)	26 (22.0)	0.185
Inotrope requirement	34 (11.8)	22 (12.9)	12 (10.2)	0.473
Requiring intubation	36 (12.5)	25 (14.7)	11 (9.3)	0.174
Requiring CABG	7 (2.5)	5 (3.2)	2 (1.7)	0.442
Length of stay, days	6 (7)	6 (8)	5 (5)	0.226
LVEF on discharge, %	46 (12)	44 (13)	49 (10)	**0.002**
**Angiographic characteristics**				
Radial access	210 (73.0)	128 (75.3)	82 (70.1)	0.451
Multivessel disease	140 (48.6)	85 (50.0)	55 (46.6)	0.571
Number of stents				0.616
0	36 (19.3)	30 (19.7)	6 (17.1)	
1	120 (64.2)	95 (62.5)	25 (71.4)	
2	26 (13.9)	22 (14.5)	4 (11.4)	
3	5 (2.7)	5 (3.3)	0	
Door-to-balloon time, minutes	88 (145)	96 (172)	80 (103)	0.390
*Discharge medications*
Aspirin	269 (93.4)	160 (94.1)	109 (92.4)	0.557
P2Y12 inhibitor	281 (97.6)	170 (100)	111 (94.1)	**0.001**
Oral anticoagulation	13 (4.5)	8 (4.7)	5 (4.2)	0.851
Betablocker	231 (82.5)	136 (84.0)	95 (80.5)	0.454
ACEI/ARB	191 (68.2)	113 (69.8)	78 (66.1)	0.517
Statin	269 (93.7)	157 (92.9)	112 (94.9)	0.488
Guideline-directed medical therapy	220 (76.4)	131 (77.1)	83 (75.4)	0.748

*Categorical data presented as n (%). Continuous data presented as mean values (standard deviation)*.

*ACEI, angiotensin converting enzyme inhibitor; ARB, angiotensin receptor blocker; CABG, coronary artery bypass graft; CAD, Coronary artery disease; LVEF, left ventricular ejection fraction; MI, myocardial infarction; PCI, percutaneous coronary intervention; STEMI, ST segment elevation myocardial infarction*.

*Statistically significant P values are highlighted in bold*.

### Telemedicine, Guideline-Directed Medical Therapy, Target Drug Dose Intensity, and Cardiovascular Risk Factor Control

The characteristics of study participants during follow-up are described in Table 2. The average number of physical consultations per patient over a 1-year period during the pandemic was lower than that in the pre-pandemic era (2.1 vs. 2.6 visits per patient per year, respectively, *p* = 0.043). Conversely, there was higher average number of teleconsultations per patient during the pandemic compared to the pre-pandemic era over the 1-year follow-up (1.2 vs. 0.2 teleconsultations per patient per year, respectively, *p* = 0.001). Cardiac rehabilitation visits were fewer during the pandemic compared to pre-pandemic era (mean of 0.1 vs. 0.3 per patient per year, respectively, *p* < 0.001).

**Table 2:** Characteristics of study participants with ST-segment elevation myocardial infarction during 1-year follow-up based on pre-pandemic or pandemic era.

	**Total (***n*** = 288)**	**Pandemic (***n*** = 170)**	**Pre-pandemic (***n*** = 118)**	***P*** **-value**
**Outpatient consultations**				
Total consultations	3.6 (3.1)	4.1 (3.5)	2.7 (2.2)	**<0.001**
Physical consultations	2.4 (1.7)	2.1 (1.6)	2.6 (1.7)	**0.043**
Teleconsultations	0.8 (1.7)	1.2 (1.9)	0.2 (1.1)	**0.001**
Cardiac rehabilitation	0.17 (0.74)	0.1 (0.7)	0.3 (0.7)	**<0.001**
Remote vital signs monitoring	97 (33.7)	59 (34.7)	38 (32.2)	0.659
**Reported symptoms**				
Typical chest pain	3 (1.2)	1 (0.7)	2 (1.9)	0.386
Atypical chest pain	22 (8.7)	12 (8.2)	10 (9.3)	0.741
Dyspnoea	21 (8.3)	10 (6.8)	11 (10.3)	0.320
Palpitations	3 (1.2)	3 (2.0)	0	0.137
Orthopnoea/PND/lower limb oedema	30 (11.8)	20 (13.6)	10 (9.3)	0.299
**Post-discharge medications**				
Aspirin	269 (93.4)	160 (94.1)	109 (92.4)	0.557
P2Y12 inhibitor	251 (87.8)	154 (90.6)	97 (83.6)	0.077
Oral anticoagulation	22 (7.6)	12 (7.1)	10 (8.5)	0.656
Beta-blocker	220 (76.9)	136 (80.0)	84 (72.4)	0.135
ACEI/ARB	202 (70.6)	126 (74.1)	76 (65.6)	0.117
Statin	263 (92.0)	158 (92.9)	105 (90.5)	0.459

*Categorical data presented as n (%). Continuous data presented as mean values (standard deviation)*.

*ACEI, angiotensin converting enzyme inhibitor; ARB, angiotensin receptor blocker; PND, paroxysmal nocturnal dyspnea*.

*Total consultations include physical and teleconsultations*.

*Statistically significant P values are highlighted in bold*.

During follow-up, all first visit post-myocardial infarction clinic consultations were physical consultations. The mean duration from discharge to first physical consultation was longer during the pandemic compared to pre-pandemic era (50 ± 39 vs. 39 ± 31 days, respectively, *p* = 0.005), with also longer mean duration between the first physical consultation to second physical consultation during the pandemic compared to pre-pandemic era (128 ± 84 vs. 98 ± 67 days, respectively, *p* = 0.008). There was no statistical difference in the uptake of remote vital signs monitoring between both study groups.

The pandemic era observed a significantly greater proportion of patients being on guideline-directed medical therapy (75.9%) compared to the pre-pandemic era (61.6%, *p* = 0.010) on follow-up. There was a trend towards achieving medication target doses in both β-blocker (19.4 vs. 15.3%, respectively, *p* = 0.363) and ACEI/ARB (9.5 vs. 5.9%, respectively, *p* = 0.278) during the pandemic compared to the pre-pandemic era.

**Figure 1:**
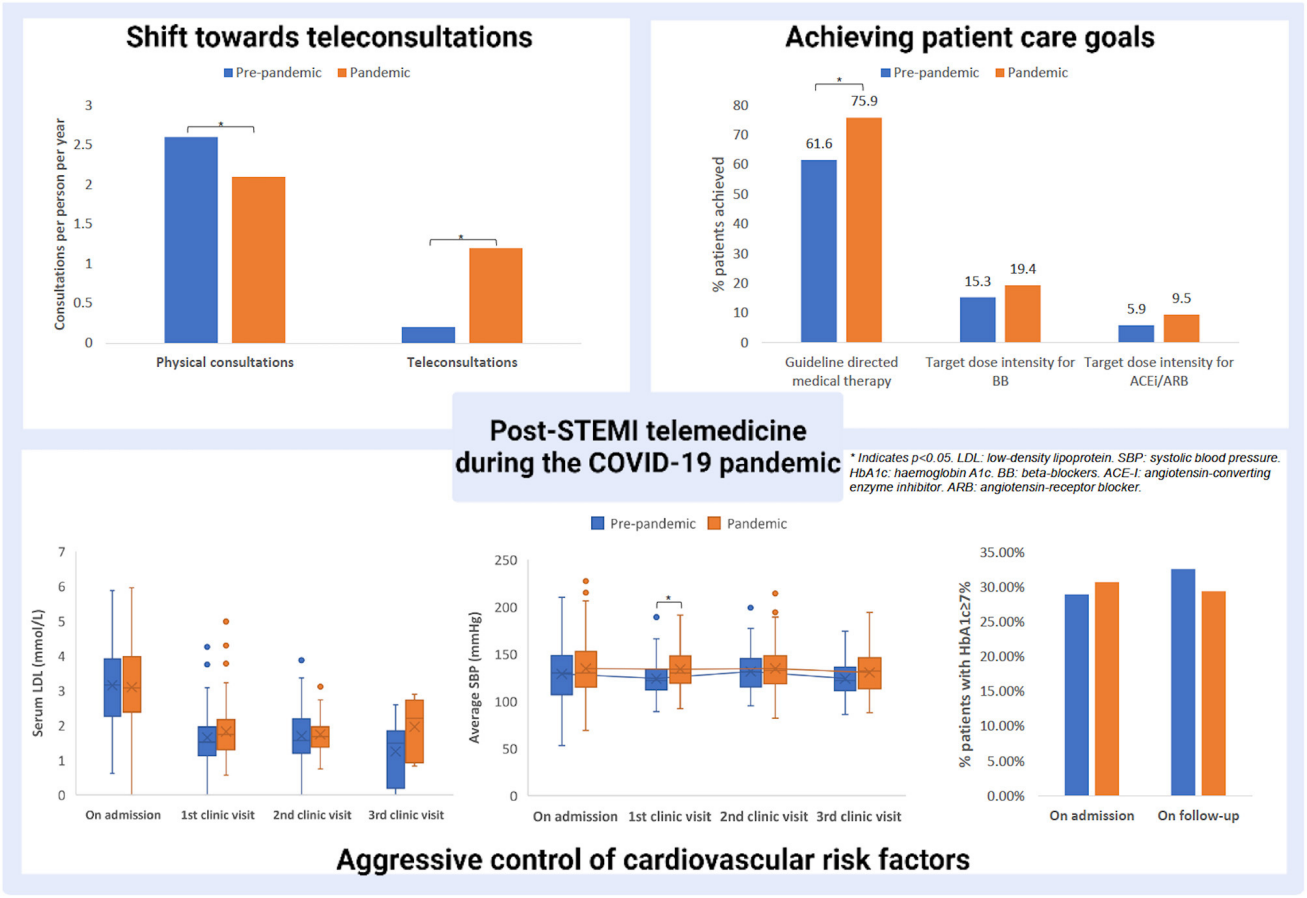
Strategies in post-STEMI care and the emergence of telemedicine during the pandemic. * indicates p < 0.05.

We observed some differences in cardiovascular risk factor control and laboratory measurements from admission to outpatient surveillance between the pandemic and pre-pandemic periods. Firstly, LDL during index admission was similar in both pandemic and pre-pandemic groups (3.09 vs. 3.14 mmol/L, respectively, *p* = 0.588). Throughout the 1-year follow-up, similar improvement in LDL was achieved in the pandemic and pre-pandemic groups on the first clinic visit (1.81 vs. 1.52 mmol/L, respectively, *p* = 0.124), second visit (1.73 vs. 1.69 mmol/L, respectively, *p* = 0.788) and third visit (1.95 vs. 1.24 mmol/L, respectively, *p* = 0.179). Secondly, the percentage of patients with Hba1c ≥7% was similar between the pandemic and pre-pandemic eras during admission (30.7 vs. 29.0%, respectively, *p* = 0.536) and first clinic visit (29.4 vs. 32.6%, respectively, *p* = 0.734). Thirdly, the average systolic blood pressure measured on discharge (134 vs. 128 mmHg, *p* = 0.09) and first clinic visit (133 vs. 123 mmHg, *p* < 0.001) was higher during the pandemic vs. the pre-pandemic eras; however such difference was no longer observed subsequently during the second (132 vs. 131 mmHg, *p* = 0.235) and third visit (130 vs. 123 mmHg, *p* = 0.174). These findings are summarized in Figure 1.

### Study Safety End-Point

The 1-year all-cause mortality rates were similar between both groups (*p* = 0.454). However, there was an overall increased cardiac readmissions in the pandemic vs. the pre-pandemic era (14.1 vs. 5.1%, *p* < 0.001). There were increased heart failure readmissions in the pandemic (7.1%) compared to pre-pandemic era (1.7%, *p* = 0.037). No differences in unplanned revascularisation (*p* = 0.787), non-fatal MI (*p* = 0.336), arrhythmia (*p* = 0.239), unstable angina (*p* = 0.701) and MACE (*p* = 0.112) were observed between the two groups (Table 3).

**Table 3:** Safety and efficacy end-points of the study population during 1-year follow-up post-index ST-segment elevation myocardial infarction admission.

	**Total (***n*** = 288)**	**Pandemic (***n*** = 170)**	**Pre-pandemic (***n*** = 118)**	***P*** **-value**
**Safety end-point**				
All-cause mortality	29 (10.1)	19 (11.2)	10 (8.5)	0.454
Cardiac readmission				
Unplanned revascularisation	3 (1.0)	2 (1.2)	1 (0.8)	0.787
Non-fatal MI	5 (1.7)	4 (2.4)	1 (0.8)	0.336
Heart failure	14 (4.9)	12 (7.1)	2 (1.7)	**0.037**
Arrythmia	2 (0.7)	2 (1.2)	0	0.239
Unstable angina	6 (2.1)	4 (2.4)	2 (1.7)	0.701
Major adverse cardiac events	59 (20.4)	43 (25.2)	16 (13.6)	0.112
**Efficacy end-point**				
Guideline-directed medical therapy	202 (70.2)	129 (75.9)	72 (61.6)	**0.010**
Achieving target dose intensity				
ACEI/ARB	23 (8.0)	16 (9.5)	7 (5.9)	0.278
Beta-blocker	51 (17.7)	33 (19.4)	18 (15.3)	0.363

*Categorical data presented as n (%)*.

*ACEI, angiotensin converting enzyme inhibitor; ARB, angiotensin receptor blocker; MI, myocardial infarction*.

*Statistically significant P values are highlighted in bold*.

On the multivariable Cox regression analysis, there was no significant association between teleconsultation and 1-year MACE [adjusted hazards ratio [aHR] 1.938, 95% confidence interval [CI] 0.896–4.190, *p* = 0.093]. Patients who achieved medication target doses (aHR 0.387, 95% CI 0.164–0.915, *p* = 0.031) and guideline-directed medical therapy (aHR 0.271, 95% CI 0.134–0.548, *p* < 0.001) were significantly associated with decreased rates of MACE after adjusting for important confounders (Table 4). There was also no significant association between teleconsultation and 1-year all-cause mortality (aHR 0.867, 95% CI 0.203–3.706, *p* = 0.847) after adjusting for important confounders (Supplementary Material 1)

**Table 4:** Cox regression for 1-year MACE in patients who survived index admission of STEMI.

**Variables**	**Adjusted hazards ratio (95% confidence ratio)**	***p*** **-value**
Teleconsultation	1.938 (0.896–4.190)	0.093
Achieving medication target doses	0.387 (0.164–0.915)	**0.031**
Post-discharge guideline-directed medical therapy	0.271 (0.134–0.548)	**<0.001**
Remote vital signs monitoring	0.512 (0.216–1.213)	0.128
Age	1.024 (1.000–1.050)	0.055
Diabetes mellitus	1.369 (0.706–2.655)	0.353
Chronic kidney disease	3.057 (1.291–7.238)	**0.011**
Left ventricular ejection fraction	0.944 (0.921–0.969)	**<0.001**
Smoker/Ex-smoker	0.609 (0.297–1.246)	0.174
Out-of-hospital cardiac arrest/cardiogenic shock	0.842 (0.348–2.037)	0.702
Admission in pandemic era	1.905 (0.827–4.390)	0.130

*Statistically significant P values are highlighted in bold*.

In addition, the association between telemedicine and guideline-directed medical therapy or medication target doses was explored. *Post-hoc* multivariable logistic regression demonstrated that having teleconsultations was significantly associated with achieving guideline-directed medical therapy [odds ratio [OR] 3.472, 95% CI 1.537–7.843, *p* = 0.003] but not achieving medication target doses (OR 1.272, 95% CI 0.636–2.542, *p* = 0.496), after adjusting for important confounders.

## Discussion

The conventional post-STEMI care has been drastically affected by the COVID-19 pandemic, and healthcare institutions have been required to adapt quickly to the stringent infectious control measures without compromising STEMI care. To our knowledge, this study is the first to systematically examine real-world data of the impact of COVID-19 pandemic on the standard of follow-up care and outcomes of STEMI patients over the ensuing year following hospital discharge. Our study has revealed several important findings. Firstly, despite exclusion of those who died while inpatient, patients admitted with STEMI during the pandemic had worse in-hospital outcomes such as increased rates of sepsis, new onset atrial fibrillation, heart failure and reduced left ventricular ejection fraction, compared to the pre-pandemic counterparts. Yet, during the 1-year follow-up, both these groups of patients had similar rates of all-cause mortality, but there were more frequent overall cardiac readmissions and heart failure readmission among those admitted during the pandemic era. This was in conjunction with the wider adaptation of teleconsultations, albeit a reduction of physical consultations, during the pandemic. Secondly, there were significantly more patients achieving guideline-directed medical therapy during the pandemic compared to the pre-pandemic era. Thirdly, there was also a trend toward increased rate of achieving medication target doses of β-blocker and ACEI/ARB therapy during the pandemic vs. the pre-pandemic era. Despite this, patients in the pandemic era had substantially higher mean LDL levels on follow-up, albeit statistically non-significant, than those in the pre-pandemic era. Fourthly, for patients who survived the index STEMI admission, achieving medication target doses and guideline-directed medical therapy during the follow-up were independently associated with a lower 1-year MACE. Even though teleconsultation was not an independent predictor of MACE, our findings highlight that teleconsultation had a significant association with achieving guideline-directed medical therapy during follow-up.

As demonstrated by a recent meta-analysis on the global impact of the COVID-19 pandemic on STEMI care (3), short-term STEMI outcomes have been shown to be unfavorable with delayed symptom onset-to-door time, door-to-balloon time, lower LVEF on discharge, suboptimal reperfusion following PCI, increased duration of intensive care unit stay and increased in-hospital mortality during the pandemic era compared to the pre-pandemic era. Similarly, our study has shown worse STEMI metrics during the index admission even after excluding those who did not survive. There were higher overall cardiac related readmissions, particularly heart failure readmissions, in the pandemic compared to pre-pandemic eras. The study sample size, however, might be too small to detect small significant differences in readmission rates in the other subgroups. Despite this, our findings revealed similar 1-year follow-up mortality between both groups. Moreover, achieving medication target doses and guideline-directed medical therapy during follow-up are independent predictors of reducing the risk of MACE. Whether teleconsultation affects the overall outcome of patients with STEMI remains to be investigated. However, it allows for safer and regular follow-up during the pandemic, with drug optimisation for patients in the early post-STEMI period. Importantly, as demonstrated by the present study that patients admitting during the pandemic had worse clinical outcomes during the index admission, this could have increased the demand for closer outpatient surveillance with increased teleconsultations particularly for patients with worse severity of cardiac disease. This might be reflected by the large standard deviation of the average number of teleconsultations in this study. Telemedicine indeed offers a synergistic avenue, in conjunction with physical consultations, in enhancing more frequent surveillance which is particularly important during the pandemic whilst maintaining the stringent infection control measures. Beyond the pandemic, teleconsultation has been shown to be cost-effective particularly for patients with myocardial infarction, as this important window of follow-up helps ameliorate adverse post-STEMI remodeling, and reduces the potential for the detrimental consequences of chronic heart failure (13).

Our recent published data displayed an increase in STEMI cases during the pandemic compared to the pre-pandemic era, which was partly due to the our regional STEMI network strategy in centralizing primary PCI service at our hospital, taking advantage of the geographical proximity of healthcare hospitals within the West of Singapore allowing timely inter-hospital transfers (14). Our previous study (4) also demonstrated that no significant door-to-balloon delay in inter-hospital transfers between the pandemic and pre-pandemic periods. This allowed the other hospitals to divert resources in providing care for the COVID-19 cases. Moreover, patients admitted during the pandemic had higher incidence of heart failure, sepsis, atrial fibrillation and lower LVEF, compared to those in the pre-pandemic period, which might play a role on the follow-up requirements during the pandemic.

Although telemedicine is a viable alternative, it is not a complete replacement for physical face-to-face consultations. In our study, there was increased overall cardiac related and heart failure readmissions during the pandemic compared to the pre-pandemic era. This could partly be due to the increased in-hospital complications during the pandemic era, such as increased prevalence of Killip class 3 heart failure at presentation and lower LVEF, compared to the pre-pandemic era. However, one might speculate that this observation suggests the limitation of teleconsultation follow-up when it comes to patients at risk of heart failure especially during the early stage following STEMI since it is limited by the absence of face-to-face clinical examination of fluid status and the lack of traditional parameter measurements in clinics such as body weight (15, 16). Nevertheless, the increasing evidence for telemedicine in heart failure management appears promising with several reviews demonstrating significant reduction in heart failure-related hospital admission compared to the conventional care (17–21). Various trials including Telemedical Interventional Monitoring in Heart Failure (TIM-HF I and TIM-HF II) have shown improved patient education, medication adherence rates, lower mortality, overall hospital admissions and heart failure admissions, with improved quality of life for patients and reduced healthcare costs with the use of telemedicine (22, 23). Hence, patients might require closer monitoring during early stage following STEMI especially those with unfavorable risk factors such as lower LVEF (10).

Teleconsultations allow rapid titration of guideline-directed medical therapy and the increased likelihood of achieving medication target doses. Despite the restrictions during the pandemic, patients were more likely to be on guideline-directed medical therapy with similar medication target dose intensities, compared to their pre-pandemic counterparts. Our study echoes previous landmark trials such that patients achieving guideline-directed medical therapy and target doses have significantly lower rate of MACE (24, 25), especially in the setting of reduced LVEF. Several reviews demonstrated significant benefits in telemedicine for HbA1c (26, 27) and LDL reductions (28, 29), although the evidence for telemedical interventions on lowering blood pressure and body mass index remains mixed (30–32). However, our study highlights the concerns regarding aggressive cardiovascular risk factor control during the pandemic. Even though we demonstrated non-significant differences between pandemic and pre-pandemic groups in terms of LDL control over the 1-year follow-up period, the absolute differences between the serial LDL levels are clinically significant. Clinicians need to be aware of the potentiality of inadequate cardiovascular risk factor control particularly in the pandemic when lifestyle and diet might be changed during the lockdown. Teleconsultation remains the cornerstone of post-STEMI care during the pandemic with timely consultations, prompt initiation and titration of optimal medical therapy, whilst ensuring social distancing and reducing the patient's exposure to the hospital. As it will take time for the telemedicine program to adapt and evolve with the dynamic demands of the pandemic, it is a possibility that there might be variations in follow-up efficacy and efficiency within each of the study groups. However, given the small study sample size, the correlation of monthly variations with clinical outcomes is likely to be underpowered to draw any conclusions. Nevertheless, these are invaluable lessons that we should take beyond the pandemic in reducing waiting and traveling time, and clinic delays, whilst maintaining the standard of post-STEMI care (33–35). The institution is constantly evolving its telemedicine programmes in conjunction with regular physical consultations, and also integrating allied health care practitioner-led remote intensive management in addition to the cardiologist-led standard care (10).

Further studies are needed to evaluate patient's perspective and potential hurdles of telemedicine. Potential hurdles to implementation of telemedicine include patient-related factors associated with older age, low health literacy, cognitive dysfunction, privacy and security concerns (6, 36, 37). In the face of constant evolution of modalities to deliver digital healthcare, the European Society of Cardiology recommends the development of specific training programs for patients, caregivers and medical staff to assist them in understanding the capabilities and limitations of telemedicine (36).

## Clinical Implications

With enhanced pandemic control measures, there is a pressing need to reduce physical consultations. Telemedicine plays an important role during the pandemic to bridge this gap in providing adequate follow-up to ensure optimisation of medical therapy post-STEMI and maintaining intensive cardiovascular risk factor control (21). It has, at least in part, contributed to the comparable 1-year post-STEMI outcomes between the pandemic and pre-pandemic eras among our patients, despite the adverse in-hospital STEMI metrics observed during the pandemic. These lessons from the pandemic serve a vital and broader role for the future with the emergence of telemedicine in post-STEMI care.

## Limitations

Although this study is the first to examine the feasibility, efficacy, and safety of telemedicine in post-STEMI care during the pandemic, our study has several limitations that merit consideration. Firstly, this is a single-center retrospective observational study with a small sample size, and hence it is not possible to infer causality between telemedicine and the observed clinical outcomes. Nevertheless, our study offers real-world data based on consecutive patients enrolled in our STEMI database, and it reflects the actual follow-up processes that transitioned from physical consultations to teleconsultations during the pandemic. Secondly, the care provided to our control group (pre-pandemic group) might not be representative of care standard that was in line with the current recommendations. Nevertheless, it was chosen as it was the most recent period possible during which the 1-year follow-up care was not affected by the pandemic. Thirdly, teleconsultation was not standardized across all attending physicians and follow-up intervals varied among the patients given the nature of the study and resource constraints during the pandemic. Fourthly, the general attitudes to health and the stresses faced by patients and healthcare providers may also differ during pre-pandemic and pandemic era. For example, the pandemic might motivate the adoption of healthier lifestyle and healthier choices; on the other hand, the social distancing and compulsory home isolation may compel a more sedentary lifestyle (38). New challenges for healthcare providers during the pandemic include the need to comply to social distancing while ensuring the rapport with patients and quality of care are not compromised, and also identifying patients at higher risk of complications in a remote setting (39). However, this study was not designed to evaluate these additional factors which might have an impact on clinical outcomes and cardiovascular risk factor control.

However, our study findings represent actual clinical practice based on the physician's clinical judgment and discretion. Moreover, telemedicine consists of both virtual telehealth clinics and the utility of digital healthcare technologies. However, our study was not designed to evaluate the deliverance of digital healthcare. Overall, the results of the study need to be interpreted with caution, as the study observations might be related to the complex interplay between the COVID-19 pandemic, telemedicine and other non-measurable factors. This retrospective cohort provides, for the first time, real-world data of the dynamic change in hospital follow-up processes in STEMI follow-up with drastic decrease in physical consultations due to social distancing policies and the rapid emergence of telemedicine. With the inherent limitations of a real-world cohort study in this ever-changing landscape during a pandemic, the preliminary findings shed light on the invaluable lessons of teleconsultation adaptation, but controlling for external influences of the pandemic is evidently not possible. Furthermore, we were not able to evaluate if the number of total consultations correlated with improvement in outcomes as the number of consultations was determined by both the routine follow-up as well as the patient's individual need for closer surveillance.

## Conclusion

Despite the unfavorable in-hospital STEMI metrics of patients admitted during the pandemic, their 1-year mortality rate was similar to those admitted during the pre-pandemic era. The pandemic led to wider adaptation of teleconsultation which might partly contribute to increased use of guideline-directed medical therapy and meeting medication target dosing. Guideline-directed medical therapy was associated with better outcomes regardless of telemedicine or the pandemic. Telemedicine, at its core, should not be considered a replacement of the traditional face-to-face doctor-patient interactions, but a synergistic extension of post-STEMI care. The invaluable lessons of telemedicine during the pandemic should be extended for future post-STEMI care.

## Data Availability Statement

The original contributions presented in the study are included in the article/Supplementary Material, further inquiries can be directed to the corresponding author/s.

## IRB Information

This study was approved by the local institution review board (NHG DSRB No. 2013/00442).

## Ethics Statement

The studies involving human participants were reviewed and approved by NHG DSRB No. 2013/00442. Written informed consent for participation was not required for this study in accordance with the national legislation and the institutional requirements.

## Author Contributions

All authors listed have made a substantial, direct and intellectual contribution to the work, and approved it for publication.

## Conflict of Interest

The authors declare that the research was conducted in the absence of any commercial or financial relationships that could be construed as a potential conflict of interest.

## Publisher's Note

All claims expressed in this article are solely those of the authors and do not necessarily represent those of their affiliated organizations, or those of the publisher, the editors and the reviewers. Any product that may be evaluated in this article, or claim that may be made by its manufacturer, is not guaranteed or endorsed by the publisher.
