# Supplementary material for: Post-ST-Segment Elevation Myocardial Infarction Follow-Up Care During the COVID-19 Pandemic and the Possible Benefit of Telemedicine: An Observational Study
Source: Front Cardiovasc Med. 2021 Oct 22;8:755822. doi: 10.3389/fcvm.2021.755822 (PMC8569238; doi:10.3389/fcvm.2021.755822)
Supplement: Supplementary file 1 [file Table_1.docx]

**Supplementary Material 1.** Cox regression for 1-year all-cause mortality in patients who survived index admission of STEMI.

| **Variables** | **Adjusted hazards ratio (95% confidence ratio)** | **p-value** |
| --- | --- | --- |
| Teleconsultation | 0.867 (0.203 – 3.706) | 0.847 |
| Achieving medication target doses | 1.295 (0.433 – 3.873) | 0.644 |
| Post-discharge guideline-directed medical therapy | 0.092 (0.028 – 0.305) | **<0.001** |
| Remote vital signs monitoring | 0.156 (0.019 – 1.283) | 0.084 |
| Age | 1.046 (1.007 – 1.087) | **0.019** |
| Diabetes mellitus | 2.913 (1.023 – 8.299) | **0.045** |
| Chronic kidney disease | 3.041 (0.996 – 9.289) | 0.051 |
| Left ventricular ejection fraction | 0.937 (0.905 – 0.970) | **<0.001** |
| Smoker/Ex-smoker | 0.797 (0.271 – 2.347) | 0.691 |
| Out-of-hospital cardiac arrest/cardiogenic shock | 1.147 (0.380 – 3.462) | 0.808 |
| Admission in pandemic era | 2.201 (0.636 – 7.621) | 0.213 |
